# Low-cost alternatives for the management of acute ischemic stroke in low and middle-income countries

**DOI:** 10.1016/j.amsu.2021.102969

**Published:** 2021-10-21

**Authors:** Gaurav Nepal, Jayant Kumar Yadav, Siddhartha Bhandari, Jeevan Gautam, Bikram Prasad Gajurel

**Affiliations:** Department of Internal Medicine, Maharajgunj Medical Campus, Tribhuvan University Institute of Medicine, Kathmandu, 44600, Nepal; Department of Internal Medicine, Maharajgunj Medical Campus, Tribhuvan University Institute of Medicine, Kathmandu, 44600, Nepal; Department of Internal Medicine, Maharajgunj Medical Campus, Tribhuvan University Institute of Medicine, Kathmandu, 44600, Nepal; Department of Internal Medicine, Maharajgunj Medical Campus, Tribhuvan University Institute of Medicine, Kathmandu, 44600, Nepal; Department of Neurology, Maharajgunj Medical Campus, Tribhuvan University Institute of Medicine, Kathmandu, 44600, Nepal

**Keywords:** Low-cost, Affordable, Economical, Inexpensive, Tenecteplase, Low dose alteplase, Minimally equipped stroke unit

## Abstract

Acute ischemic stroke (AIS) patients arriving within a suitable time frame are treated with recanalization therapy i.e. intravenous thrombolysis (IVT) with alteplase and/or mechanical thrombectomy (MT). IVT with alteplase is indicated in AIS patients presenting within 4.5 hours of onset regardless of vascular territory involved. MT is indicated in AIS patients presenting within 24 hours of onset with large vessel occlusion in the anterior circulation. However, MT is ludicrously expensive and requires exorbitant setup, devices, and expertise which is not currently feasible in LMICs. Therefore, in LMICs the only feasible recanalization option left for AIS patients is IVT. The cost of IVT varies across the LMICs, however, most of them cost around 2000–5000 USD. Apart from IVT, patients with AIS often have other significant medical costs including those for neuroimaging, intensive care, and prolonged rehabilitative treatment. In LMICs, these costs can only be afforded by a handful of patients. The majority of the LMICs have health insurance in their infancy and family members of AIS patients opt-out IVT due to the economic burden. In general, the current treatment guidelines for AIS are not very useful in LMICs because of cost-related issues among several other factors. In this editorial, we discuss evidence for alternative treatment strategies that can help tackle the rising epidemic of AIS in poor countries by improvising on existing clinical guidelines and seeking alternative treatment regimens.

## 1 Introduction

Stroke is the second leading cause of global death and the third leading cause of premature death and disability as measured in Disability Adjusted Life Years (DALY) by the Global Burden of Disease Study. Low-and middle-income countries (LMICs) share the major burden of stroke comprising 75% of deaths from stroke and 81% of stroke-related DALYs [1]. Patients from LMICs get stroke 15 years earlier than those in high-income countries and stroke affects individuals in the most productive phase of their lives [1]. Further, stroke incidence and mortality rates have been correlated with national per capita income. A study of 56 registries worldwide showed that there was a 42% decrease in stroke incidence in high-income countries, whereas LMICs experienced a 100% increase in stroke incidence [2].

For a patient presenting with signs and symptoms suggestive of stroke, non-contrast Computed Tomography (CT) is typically the first diagnostic study done to rule out intracerebral hemorrhage and distinguish acute ischemic stroke (AIS) from hemorrhagic stroke. If CT does not show hemorrhage and the patient has arrived within a suitable time frame, the patient might be a candidate for recanalization therapy [3]. While this is the current standard of care as per the American Stroke Association and other leading bodies’ recommendations, the reality is different for resource-poor rural settings in Asia and Africa. According to 2017 WHO data on the availability of medical devices, 21% of LMICs still do not have a CT scanner [4]. Even if available, these are expensive and unaffordable for many, especially in LMICs where insurance schemes are virtually non-existent. Despite AIS accounting for up to 80–85% of total stroke cases, it is difficult to distinguish from hemorrhagic stroke without imaging. Moreover, even if a hemorrhage is ruled out, recanalization therapy is expensive and unaffordable for the majority of the population. In general, the current treatment strategies for stroke are not very useful in resource-poor settings because of cost-related issues among several other factors. In this editorial, we discuss currently available evidence for alternative treatment strategies that can help tackle the rising epidemic of stroke in poor countries by improvising on existing clinical guidelines and seeking alternative treatment regimens.

### 1.1 Establishment of minimally equipped stroke units

As discussed earlier, imaging facilities and stroke treatment centers are more of an exception than the norm in LMICs due to accessibility and cost parameters. Although neurological deterioration due to lack of recanalization occurs during the first hours, the main factors leading to stroke mortality are complications like aspiration pneumonia, cardiac and thromboembolic disease. This is where stroke units (SUs) make a difference. SUs refers to organized inpatient care for stroke patients, provided by a multidisciplinary team specialized in stroke management. This includes medical, nursing, physiotherapy, speech therapist, and social work staff operating within a discrete hospital ward. SU aims to improve diagnostic accuracy, prevention of complications through specialized nursing care, appropriate monitoring as well as early rehabilitation (5). Evidence suggests that acute stroke patients have a better outcome when admitted to a dedicated stroke unit (SU) than when admitted to conventional units. SUs have been shown to significantly reduce disability and mortality, even in the era when brain imaging was unavailable. This suggests that SUs can be a viable option in LMICs.

Ideally, stroke units are highly specialized and have multidisciplinary team, stroke-trained nurses, brain CT scan 24/7, CT priority for stroke patients, extracranial Doppler sonography, automated electrocardiographic monitoring, intravenous rt-PA protocols etc. [5]. However, all of these may not be available in LMICs. Minimally equipped stroke units (MESU) are viable options in such places. In a study conducted in Guinea in Africa, mortality was significantly lower in the patients receiving treatment in a MESU compared to those receiving treatment in the normal ward (7.2 vs. 22.3%, *p* < 0.0001) as well as medical complications including frequency of UTIs, bedsores, and pneumonia. Patients treated in MESU also had better post-treatment modified Rankin Scale (mRS) and National Institute of Health Stroke Scale (NIHSS) scores. The MESU had three *acute* beds, separated from other neurology wards, and were equipped with monitors for heart rate, blood pressure, and oxygen saturation, and a portable oxygen concentrator. Patients were evaluated every 4 hours for clinical parameters, body temperature, and NIHSS by a dedicated stroke team that consisted of neurologists, neurologists in training, nurses, and three physiotherapists. Similar results were obtained from a study in South Africa [6,7].

It is worth mentioning that many earlier trials of stroke units were conducted when access to CT scans was limited [[8], [9], [10], [11]]. In these trials, the diagnosis was largely based on clinical assessment and basic investigations. Despite these limitations, the reduction in adverse outcomes of death or dependency in patients treated in stroke units was very similar to that of the systematic review by Stroke Units Trialists' Collaboration, which included studies on specialized stroke units [12]. This is important because the imaging facilities are not widely available in LMICs and tend to be located in urban areas [13]. This shows that even MESU can be useful in reducing stroke-related burden in LMICs before specialized and highly specialized stroke units become a reality there.

### 1.2 Intravenous tenecteplase

Intravenous thrombolysis with alteplase has a potential benefit if started within 4.5 hours of the onset of AIS symptoms. However, the rates of thrombolysis remain low universally and more so in LMICs. Many barriers, including but not limited to, prehospital and in-hospital delay, prevent early administration of thrombolytic therapy. Superimposing barriers in LMICs include accessibility and affordability of alteplase. In a study by Nepal et al. only 20% of AIS patients reached the hospital in the window period; and among them, 35% were denied thrombolysis even after reaching the hospital within the required time frame simply because they were unable to afford alteplase [45]. Currently, alteplase is the only FDA-approved thrombolytic agent for AIS. This was approved following a landmark trial in 1996 from the National Institutes of Health and serves as the basis for the current use of alteplase for AIS [39]. Twenty-five years later, although tenecteplase is way cheaper, affordable, and has superior thrombolytic properties compared to alteplase, alteplase remains the preferred thrombolytic agent.

An ideal thrombolytic agent should be effective and safe, fast and long-acting, active in platelet-rich thrombi, have a long window period for administration, and have high fibrin specificity. Alteplase is far from ideal for meeting these requirements. Further, alteplase is associated with an increased risk of hemorrhage [14]. Tenecteplase was bioengineered to overcome these limitations. Although tenecteplase has been FDA approved for use in patients with acute myocardial infarction following the ASSENT trial, it has not been so for AIS [15]. Tenecteplase has greater fibrin specificity and a longer half-life than alteplase. These pharmacologic differences allow tenecteplase to be administered as a bolus rather than by infusion as alteplase, making the administration of alteplase tedious. Therefore, many stroke centers involve clinical pharmacists in stroke protocols. However, this is not a feasible option in rural settings, reinforcing the vote for the usage of tenecteplase even more.

Five randomized clinical trials have compared the safety and efficacy of tenecteplase to alteplase. In these trials, tenecteplase is at least as effective or more effective than alteplase for neurologic improvement after AIS [[16], [17], [18], [19], [20]]. In the majority of studies, tenecteplase at the dose of 0.25 mg/kg was found to have early neurologic improvement and better functional outcomes at the first 24 hours and 90 days of the event in terms of disability when compared to alteplase. The tenecteplase dose of 0.4 mg/kg was found to result in higher rates of intracerebral hemorrhage. Using the results of those five randomized controlled trials, four separate meta-analyses have been performed, and none of those concluded that alteplase is superior to tenecteplase [[21], [22], [23], [24]].

As discussed, besides better efficacy and safety profile, tenecteplase is cheaper, easier to administer, and may have fewer bleeding complications than alteplase. Thus physicians in LMICs should consider tenecteplase as an alternative to the standard alteplase in patients with AIS.

### 1.3 Intravenous low dose alteplase

The standard recommended dose of alteplase used in AIS is 0.9 mg/kg derived from studies based on the American and European populations. This dosage has not been adequately tested in Asian communities. The Japan Alteplase clinical trial conducted in 2006 compared the safety and efficacy of low dose alteplase (0.6 mg/kg body weight; maximum 60 kg) to the standard dose (0.9 mg/kg; maximum 90mg). In patients receiving 0.6 mg/kg alteplase, the outcome and the incidence of symptomatic intracerebral hemorrhage (SICH) were comparable to published data for 0.9 mg/kg, indicating that alteplase, when administered at 0.6 mg/kg to Japanese patients, might offer a clinical efficacy and safety that is comparable to that reported in North America and the European Union for a 0.9 mg/kg dose [25].

In another Japanese study, the rates of early recanalization and favorable outcomes provoked by low dose alteplase were comparable to that previously reported with the standard dose in 58 patients. None of the patients had symptomatic intracranial hemorrhage [26]. The ENCHANTED trial, the largest randomized trial involving predominantly Asian patients, showed that alteplase at a low dose caused a significantly lower risk of SICH. However, low dose alteplase did not show non-inferiority to standard dose in terms of death and disability at 90 days [27]. Similar results were obtained from a secondary analysis of the ENCHANTED trial. The outcomes did not differ by age, ethnicity, or severity of AIS [28]. Further, another secondary analysis using the data from the ENCHANTED trial revealed that low dose alteplase had better outcomes than standard-dose alteplase in patients treated with prior antiplatelet therapy when compared to those not using antiplatelet therapy (mRS scores of 2–6; OR 0.84; 95% CI, 0.62–1.12 versus OR 1.16; 95% CI, 0.99–1.36) However, the data did not gain statistical significance [29]. Data from the Korean ENCHANTED trial found no significant difference in favorable functional outcomes between the standard and low dose groups (39% vs. 21%; OR 2.39; 95% CI, 0.73 to 7.78; p = 0.149), although the risk of bleeding was lower in the low dose group [30]. Similar outcomes were obtained from a study in Vietnam [31].

Because of lower cost and the reduced risk of bleeding, low-dose alteplase can be an alternative option in patients with AIS. Although a low dose appears safe, whether or not does it translate into overall clinical benefit is debatable and requires head-to-head RCTs before it becomes the norm.

### 1.4 Intravenous streptokinase

Streptokinase is an alternative plasminogen activator, produced by strains of *Streptococcus*. It is commonly used in the treatment of acute coronary syndromes (ACS) and has comparable efficacy and safety to other thrombolytic agents [32,33]. Four clinical trials involving the use of Streptokinase in AIS have been published which did not demonstrate its clinical benefits. However, these trials used a fixed dose of 1.5 million units of Streptokinase as used in ACS trials (no dose escalation studies performed) and also used it in those presenting beyond 3 hours of symptoms onset. In these trials, the rate of sICH following streptokinase administration was 8–21% and was more frequent in younger patients who had received higher doses relative to their body weight [[34], [35], [36], [37]]. However, a meta-analysis of all 4 trials involving 1292 patients found that the 90 days mortality and severe disability was significantly lower in patients treated with streptokinase within 3 hours of symptom onset, than those treated after 3 hours [38]. This data provides an opportunity for considering a new streptokinase trial with strict a patient and dose selection as done in the NINDS trial of alteplase [39].

Streptokinase differs from alteplase in several ways-it is less fibrin specific, which might have contributed to the higher rates of hemorrhagic transformation in completed trials. Second, it decreases fibrinogen levels significantly accounting for higher rates of systemic bleeding. Third, it has a shorter half-life in vivo. Despite these unwanted properties, it may be justifiable to use streptokinase in LMICs due to the unavailability of alteplase, which is because of its cost. The cost of streptokinase is less than 1/10th the cost of alteplase and is widely available in these countries for the treatment of ACS. Hence, the need for an affordable alternative justifies streptokinase trials in centers where alteplase is unavailable. Further, even though streptokinase is shown to be less efficacious, the lower cost per quality-adjusted life-year would justify its use in resource-poor countries[40]. Interestingly, pegylation of truncated streptokinase offers promise as some of the derivatives showed enhanced plasma resistance, longer half-life, improved fibrin specificity, and reduced immune reactivity, thereby offsetting the drawbacks of streptokinase [41].

Streptokinase is a cheap and affordable alternative for thrombolysis for stroke in LMICs. It is important to conduct a study to determine the optimum dose of streptokinase in AIS rather than rely on the findings of ACS trials. Factors to be considered in future streptokinase trials in acute stroke include, but are not limited to, earlier administration (<3 hours since the onset of symptoms), lower doses of streptokinase (weight-adjusted doses with a maximum of 1 million units), avoidance of concomitant aspirin, and appropriate patient selection [38].

### 1.5 Intravenous reteplase

Reteplase is a second-generation non-glycosylated deletion mutant of alteplase and has been approved for use in ACS [42]. It is less fibrin-specific than alteplase but has a longer half-life, allowing the administration of double bolus IV injection. As a result of mutation, reteplase does not bind highly to fibrin; unbound Reteplase can penetrate the clot and improve in vivo fibrinolytic activity [43]. Reteplase was first tested in the experimental animal model, in which 34 rabbits were embolized using aged heterologous thrombi. Intravenous treatment with alteplase (n = 11, 6 mg/kg bolus over 1 hour), reteplase (n = 11, 1 mg/kg bolus) or placebo (n = 10) was started after 1 hour of stroke induction following DWI confirmation. Improved perfusion was seen in the alteplase and reteplase group compared to placebo using a semi-quantitative scale (p < 0.01, alteplase vs. controls; p < 0.05, reteplase vs. controls) [44]. So far, prospective human trials to evaluate the safety and efficacy of intravenous reteplase in AIS have not been conducted. Looking at the clinical benefit of reteplase in ACS, easy availability and its cheap cost, a head-to-head trial to compare the efficacy of reteplase and alteplase is necessary for LMICs. If found non-inferior to alteplase regarding safety and efficacy outcomes, this drug has enormous potential to reduce the AIS recanalization therapy in LMICs of Asia and Africa.

## 2 Conclusion

Cost-effective therapy for AIS warrants further research, especially in LMICs. Some of the aforementioned alternatives already have robust evidence for clinical use while some warrant further research or re-designing of research before they can be used clinically in AIS patients. With support from the research front, policymakers, and health authorities, the patients of LMICs afflicted with AIS may benefit significantly from timely and affordable treatment.

## Ethical approval

Not applicable.

## Sources of funding

None.

## Author contribution

Study concept and design: GN, JKY, and BPG. Data collection: GN, JG and JKY. Analysis and interpretation of data: GN and JKY. Drafting of the manuscript: JKY, GN, and SB. Supervision: BPG. All authors read and approved the final manuscript.

## Conflicts of interest

None.

## Registration of research studies

1. Name of the registry: not applicable
2. Unique Identifying number or registration ID: not applicable
3. Hyperlink to your specific registration (must be publicly accessible and will be checked): not applicable

## Guarantor

Dr. Gaurav Nepal.

## Consent

Not applicable.

## CRediT authorship contribution statement

**Gaurav Nepal:** Conceptualization, and design, Data collection, Formal analysis, Writing – original draft, of the manuscript. **Jayant Kumar Yadav:** Conceptualization, and design, Data collection, Formal analysis, Writing – original draft, of the manuscript. **Siddhartha Bhandari:** Writing – original draft, of the manuscript. **Jeevan Gautam:** Data collection. **Bikram Prasad Gajurel:** Conceptualization, and design, Supervision.
